# Supplementary material for: Modeling glioblastoma heterogeneity as a dynamic network of cell states
Source: Mol Syst Biol. 2021 Sep 16;17(9):e10105. doi: 10.15252/msb.202010105 (PMC8444284; doi:10.15252/msb.202010105)
Supplement: Supplementary file 5 — Source Data for Figure 3 [file MSB-17-e10105-s001.zip › Figure3A_sourcedata/GSEA_3065/hallmarks_state3.GseaPreranked.1623416274744/HALLMARK_PEROXISOME.html]

Details for gene set HALLMARK\_PEROXISOME[GSEA]

|  || Dataset | state3 |
| Phenotype | NoPhenotypeAvailable |
| Upregulated in class | na\_neg |
| GeneSet | HALLMARK\_PEROXISOME |
| Enrichment Score (ES) | -0.31782976 |
| Normalized Enrichment Score (NES) | -1.0607096 |
| Nominal p-value | 0.35551947 |
| FDR q-value | 0.557312 |
| FWER p-Value | 1.0 |
Table: GSEA Results Summary

  

Fig 1: Enrichment plot: HALLMARK\_PEROXISOME      
 Profile of the Running ES Score & Positions of GeneSet Members on the Rank Ordered List

  

| PROBE | GENE SYMBOL | GENE\_TITLE | RANK IN GENE LIST | RANK METRIC SCORE | RUNNING ES | CORE ENRICHMENT || 1 | TOP2A |  |  | 42 | 0.448 | 0.0514 | No |
| 2 | RDH11 |  |  | 212 | 0.277 | 0.0666 | No |
| 3 | DHCR24 |  |  | 244 | 0.265 | 0.0963 | No |
| 4 | FADS1 |  |  | 331 | 0.234 | 0.1158 | No |
| 5 | SMARCC1 |  |  | 397 | 0.219 | 0.1357 | No |
| 6 | ELOVL5 |  |  | 450 | 0.209 | 0.1560 | No |
| 7 | FDPS |  |  | 518 | 0.196 | 0.1729 | No |
| 8 | MSH2 |  |  | 885 | 0.150 | 0.1495 | No |
| 9 | HSD17B11 |  |  | 1083 | 0.132 | 0.1434 | No |
| 10 | CLN6 |  |  | 1132 | 0.127 | 0.1538 | No |
| 11 | HSD17B4 |  |  | 1193 | 0.122 | 0.1622 | No |
| 12 | CLN8 |  |  | 1340 | 0.112 | 0.1594 | No |
| 13 | SEMA3C |  |  | 1479 | 0.105 | 0.1567 | No |
| 14 | ACOX1 |  |  | 1544 | 0.101 | 0.1619 | No |
| 15 | LONP2 |  |  | 1707 | 0.091 | 0.1547 | No |
| 16 | CADM1 |  |  | 1735 | 0.090 | 0.1629 | No |
| 17 | GSTK1 |  |  | 2238 | 0.067 | 0.1134 | No |
| 18 | ABCC5 |  |  | 2250 | 0.066 | 0.1204 | No |
| 19 | IDI1 |  |  | 2285 | 0.065 | 0.1247 | No |
| 20 | IDH1 |  |  | 2376 | 0.062 | 0.1220 | No |
| 21 | SLC23A2 |  |  | 2399 | 0.061 | 0.1271 | No |
| 22 | CTPS1 |  |  | 2789 | 0.047 | 0.0883 | No |
| 23 | SLC35B2 |  |  | 2872 | 0.045 | 0.0845 | No |
| 24 | ABCD3 |  |  | 2974 | 0.042 | 0.0781 | No |
| 25 | IDE |  |  | 3067 | 0.039 | 0.0724 | No |
| 26 | PEX13 |  |  | 3146 | 0.037 | 0.0681 | No |
| 27 | MLYCD |  |  | 3359 | 0.032 | 0.0477 | No |
| 28 | ATXN1 |  |  | 3504 | 0.028 | 0.0346 | No |
| 29 | PEX6 |  |  | 3666 | 0.024 | 0.0191 | No |
| 30 | CDK7 |  |  | 3870 | 0.018 | -0.0020 | No |
| 31 | GNPAT |  |  | 4907 | -0.009 | -0.1201 | No |
| 32 | ERCC3 |  |  | 4956 | -0.010 | -0.1244 | No |
| 33 | HMGCL |  |  | 5049 | -0.012 | -0.1335 | No |
| 34 | CAT |  |  | 5510 | -0.024 | -0.1834 | No |
| 35 | MVP |  |  | 5610 | -0.028 | -0.1913 | No |
| 36 | ACSL4 |  |  | 5729 | -0.031 | -0.2011 | No |
| 37 | SOD2 |  |  | 5817 | -0.033 | -0.2069 | No |
| 38 | ACOT8 |  |  | 5851 | -0.034 | -0.2064 | No |
| 39 | ACAA1 |  |  | 6069 | -0.042 | -0.2261 | No |
| 40 | VPS4B |  |  | 6113 | -0.043 | -0.2256 | No |
| 41 | ACSL1 |  |  | 6134 | -0.044 | -0.2224 | No |
| 42 | BCL10 |  |  | 6249 | -0.048 | -0.2295 | No |
| 43 | ALDH9A1 |  |  | 6304 | -0.050 | -0.2295 | No |
| 44 | SIAH1 |  |  | 6359 | -0.051 | -0.2293 | No |
| 45 | ISOC1 |  |  | 6447 | -0.054 | -0.2325 | No |
| 46 | SLC25A17 |  |  | 6631 | -0.063 | -0.2457 | No |
| 47 | ECI2 |  |  | 6954 | -0.078 | -0.2729 | No |
| 48 | IDH2 |  |  | 7068 | -0.085 | -0.2753 | No |
| 49 | PEX2 |  |  | 7209 | -0.094 | -0.2796 | No |
| 50 | TSPO |  |  | 7327 | -0.102 | -0.2802 | No |
| 51 | CRAT |  |  | 7655 | -0.133 | -0.3011 | Yes |
| 52 | ECH1 |  |  | 7658 | -0.134 | -0.2845 | Yes |
| 53 | CTBP1 |  |  | 7775 | -0.148 | -0.2793 | Yes |
| 54 | ERCC1 |  |  | 7810 | -0.154 | -0.2639 | Yes |
| 55 | SCP2 |  |  | 7919 | -0.170 | -0.2550 | Yes |
| 56 | PABPC1 |  |  | 7960 | -0.179 | -0.2372 | Yes |
| 57 | SLC25A4 |  |  | 8291 | -0.256 | -0.2432 | Yes |
| 58 | CNBP |  |  | 8379 | -0.286 | -0.2173 | Yes |
| 59 | YWHAH |  |  | 8384 | -0.289 | -0.1816 | Yes |
| 60 | HRAS |  |  | 8396 | -0.293 | -0.1461 | Yes |
| 61 | FIS1 |  |  | 8506 | -0.342 | -0.1158 | Yes |
| 62 | SOD1 |  |  | 8526 | -0.349 | -0.0742 | Yes |
| 63 | PRDX5 |  |  | 8591 | -0.395 | -0.0321 | Yes |
| 64 | ITGB1BP1 |  |  | 8599 | -0.402 | 0.0175 | Yes |
Table: GSEA details [plain text format]

  

Fig 2: HALLMARK\_PEROXISOME: Random ES distribution      
 Gene set null distribution of ES for **HALLMARK\_PEROXISOME**
